# Supplementary material for: Mining Indole Alkaloid Synthesis Gene Clusters from Genomes of 53 Claviceps Strains Revealed Redundant Gene Copies and an Approximate Evolutionary Hourglass Model
Source: Toxins (Basel). 2021 Nov 13;13(11):799. doi: 10.3390/toxins13110799 (PMC8625505; doi:10.3390/toxins13110799)
Supplement: Supplementary file 1 [file toxins-13-00799-s001.zip › !Figure S2_multi-copy gene trees.pptx]

## Slide 1
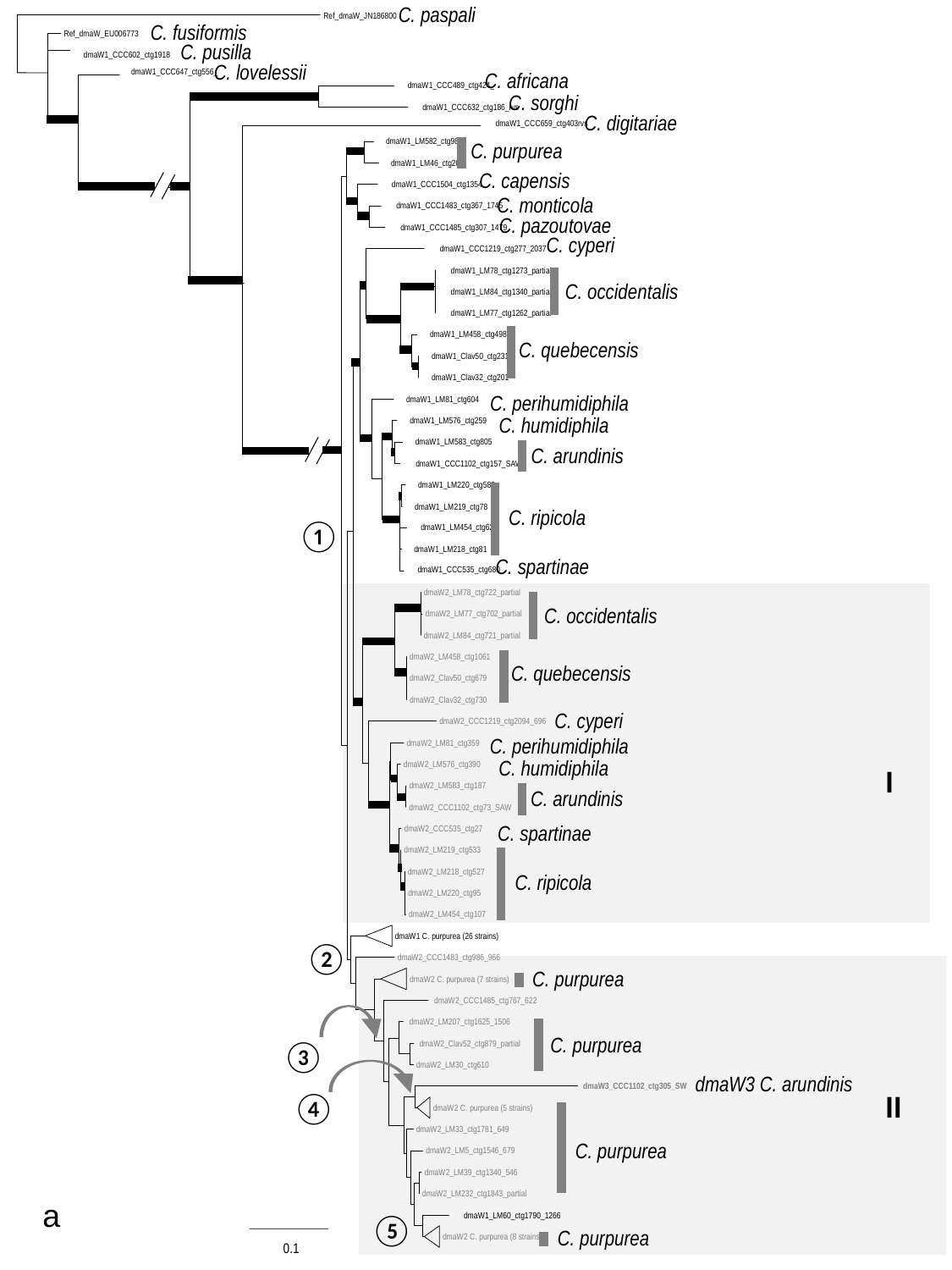

C. paspali
Ref_dmaW_JN186800
C. fusiformis
Ref_dmaW_EU006773
C. pusilla
dmaW1_CCC602_ctg1918
C. lovelessii
dmaW1_CCC647_ctg556_
C. africana
dmaW1_CCC489_ctg424_
C. sorghi
dmaW1_CCC632_ctg186_rvs
C. digitariae
dmaW1_CCC659_ctg403rvs
dmaW1_LM582_ctg98
dmaW1_LM46_ctg209
dmaW1_CCC1504_ctg1354
dmaW1_CCC1483_ctg367_1745
dmaW1_CCC1485_ctg307_1479
dmaW1_CCC1219_ctg277_2037
dmaW1_LM78_ctg1273_partial
dmaW1_LM84_ctg1340_partial
dmaW1_LM77_ctg1262_partial
dmaW1_LM458_ctg498
dmaW1_Clav50_ctg231
dmaW1_Clav32_ctg201
dmaW1_LM81_ctg604
dmaW1_LM576_ctg259
dmaW1_LM583_ctg805
dmaW1_CCC1102_ctg157_SAW
dmaW1_LM220_ctg588
dmaW1_LM219_ctg78
dmaW1_LM454_ctg623
dmaW1_LM218_ctg81
dmaW1_CCC535_ctg680
dmaW2_LM78_ctg722_partial
dmaW2_LM77_ctg702_partial
dmaW2_LM84_ctg721_partial
dmaW2_LM458_ctg1061
dmaW2_Clav50_ctg679
dmaW2_Clav32_ctg730
dmaW2_CCC1219_ctg2094_696
dmaW2_LM81_ctg359
dmaW2_LM576_ctg390
dmaW2_LM583_ctg187
dmaW2_CCC1102_ctg73_SAW
dmaW2_CCC535_ctg27
dmaW2_LM219_ctg533
dmaW2_LM218_ctg527
dmaW2_LM220_ctg95
dmaW2_LM454_ctg107
dmaW1 C. purpurea (26 strains)
dmaW2_CCC1483_ctg986_966
dmaW2 C. purpurea (7 strains)
dmaW2_CCC1485_ctg767_622
dmaW2_LM207_ctg1625_1506
dmaW2_Clav52_ctg879_partial
dmaW2_LM30_ctg610
dmaW3_CCC1102_ctg305_SW
dmaW2 C. purpurea (5 strains)
dmaW2_LM33_ctg1781_649
dmaW2_LM5_ctg1546_679
dmaW2_LM39_ctg1340_546
dmaW2_LM232_ctg1843_partial
dmaW1_LM60_ctg1790_1266
dmaW2 C. purpurea (8 strains)
C. purpurea
C. capensis
C. monticola
C. pazoutovae
C. cyperi
C. occidentalis
C. quebecensis
C. perihumidiphila
C. humidiphila
C. arundinis
C. ripicola
①
C. spartinae
C. occidentalis
C. quebecensis
C. cyperi
C. perihumidiphila
C. humidiphila
I
C. arundinis
C. spartinae
C. ripicola
②
C. purpurea
C. purpurea
③
dmaW3 C. arundinis
II
④
C. purpurea
a
⑤
C. purpurea
0.1

## Slide 2
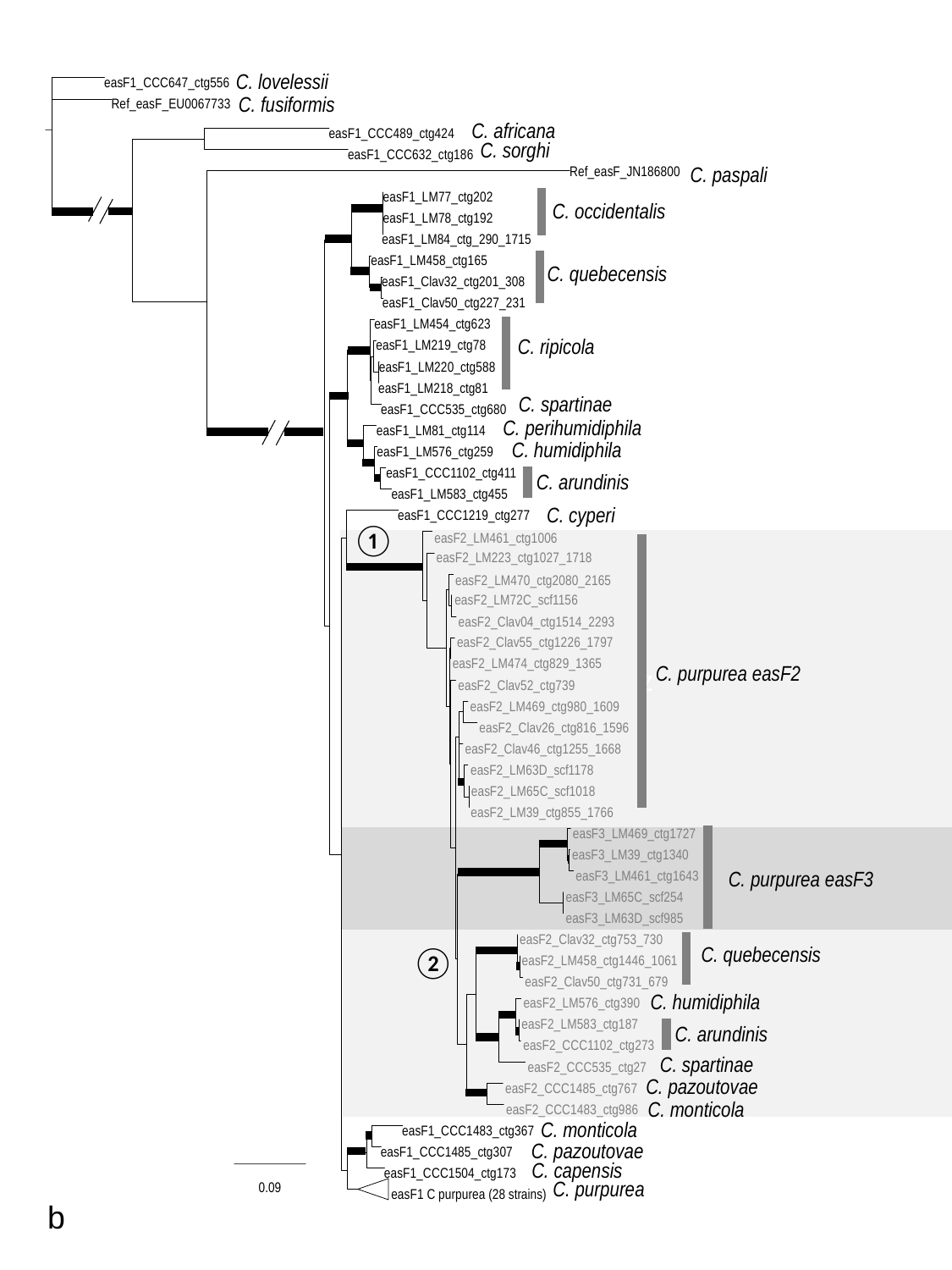

C. lovelessii
easF1_CCC647_ctg556
Ref_easF_EU0067733
C. fusiformis
C. africana
easF1_CCC489_ctg424
easF1_CCC632_ctg186
C. sorghi
C. paspali
Ref_easF_JN186800
easF1_LM77_ctg202
easF1_LM78_ctg192
easF1_LM84_ctg_290_1715
easF1_LM458_ctg165
easF1_Clav32_ctg201_308
easF1_Clav50_ctg227_231
easF1_LM454_ctg623
easF1_LM219_ctg78
easF1_LM220_ctg588
easF1_LM218_ctg81
easF1_CCC535_ctg680
easF1_LM81_ctg114
easF1_LM576_ctg259
easF1_CCC1102_ctg411
easF1_LM583_ctg455
easF1_CCC1219_ctg277
easF2_LM461_ctg1006
easF2_LM223_ctg1027_1718
easF2_LM470_ctg2080_2165
easF2_LM72C_scf1156
easF2_Clav04_ctg1514_2293
easF2_Clav55_ctg1226_1797
easF2_LM474_ctg829_1365
easF2_Clav52_ctg739
easF2_LM469_ctg980_1609
easF2_Clav26_ctg816_1596
easF2_Clav46_ctg1255_1668
easF2_LM63D_scf1178
easF2_LM65C_scf1018
easF2_LM39_ctg855_1766
easF3_LM469_ctg1727
easF3_LM39_ctg1340
easF3_LM461_ctg1643
easF3_LM65C_scf254
easF3_LM63D_scf985
easF2_Clav32_ctg753_730
easF2_LM458_ctg1446_1061
easF2_Clav50_ctg731_679
easF2_LM576_ctg390
easF2_LM583_ctg187
easF2_CCC1102_ctg273
easF2_CCC535_ctg27
easF2_CCC1485_ctg767
easF2_CCC1483_ctg986
easF1_CCC1483_ctg367
easF1_CCC1485_ctg307
easF1_CCC1504_ctg173
easF1 C purpurea (28 strains)
C. occidentalis
C. quebecensis
C. ripicola
C. spartinae
C. perihumidiphila
C. humidiphila
C. arundinis
C. cyperi
①
z
C. purpurea easF2
C. purpurea easF3
C. quebecensis
②
C. humidiphila
C. arundinis
C. spartinae
C. pazoutovae
C. monticola
C. monticola
C. pazoutovae
C. capensis
0.09
C. purpurea
b

## Slide 3
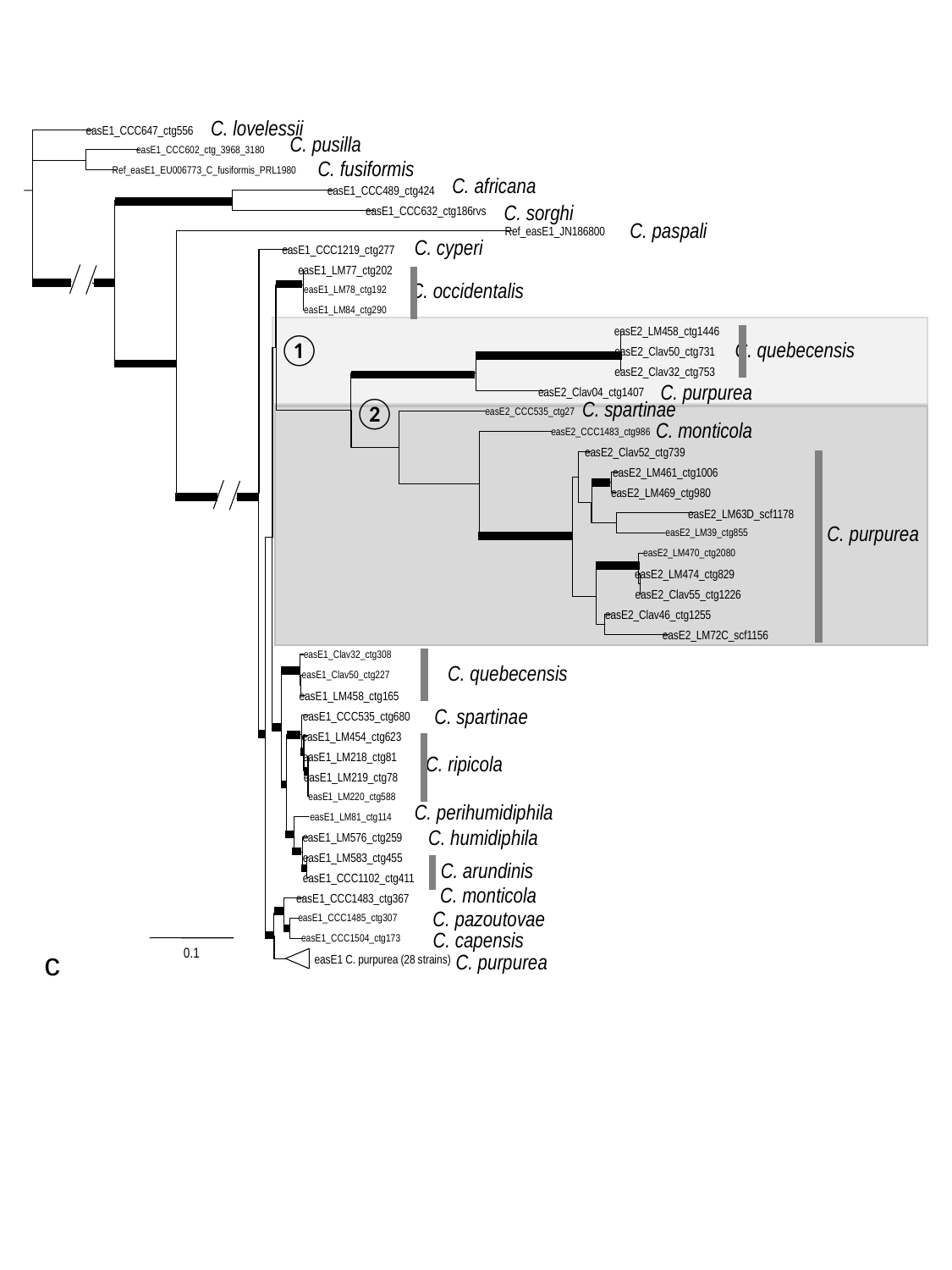

C. lovelessii
easE1_CCC647_ctg556
C. pusilla
easE1_CCC602_ctg_3968_3180
C. fusiformis
Ref_easE1_EU006773_C_fusiformis_PRL1980
C. africana
easE1_CCC489_ctg424
C. sorghi
easE1_CCC632_ctg186rvs
C. paspali
Ref_easE1_JN186800
C. cyperi
easE1_CCC1219_ctg277
easE1_LM77_ctg202
easE1_LM78_ctg192
easE1_LM84_ctg290
easE2_LM458_ctg1446
easE2_Clav50_ctg731
easE2_Clav32_ctg753
easE2_Clav04_ctg1407
easE2_CCC535_ctg27
easE2_CCC1483_ctg986
easE2_Clav52_ctg739
easE2_LM461_ctg1006
easE2_LM469_ctg980
easE2_LM63D_scf1178
easE2_LM39_ctg855
easE2_LM470_ctg2080
easE2_LM474_ctg829
easE2_Clav55_ctg1226
easE2_Clav46_ctg1255
easE2_LM72C_scf1156
easE1_Clav32_ctg308
easE1_Clav50_ctg227
easE1_LM458_ctg165
easE1_CCC535_ctg680
easE1_LM454_ctg623
easE1_LM218_ctg81
easE1_LM219_ctg78
easE1_LM220_ctg588
easE1_LM81_ctg114
easE1_LM576_ctg259
easE1_LM583_ctg455
easE1_CCC1102_ctg411
C. occidentalis
C. quebecensis
①
C. purpurea
②
C. spartinae
C. monticola
C. purpurea
C. quebecensis
C. spartinae
C. ripicola
C. perihumidiphila
C. humidiphila
C. arundinis
C. monticola
easE1_CCC1483_ctg367
C. pazoutovae
easE1_CCC1485_ctg307
C. capensis
easE1_CCC1504_ctg173
c
0.1
C. purpurea
easE1 C. purpurea (28 strains)

## Slide 4
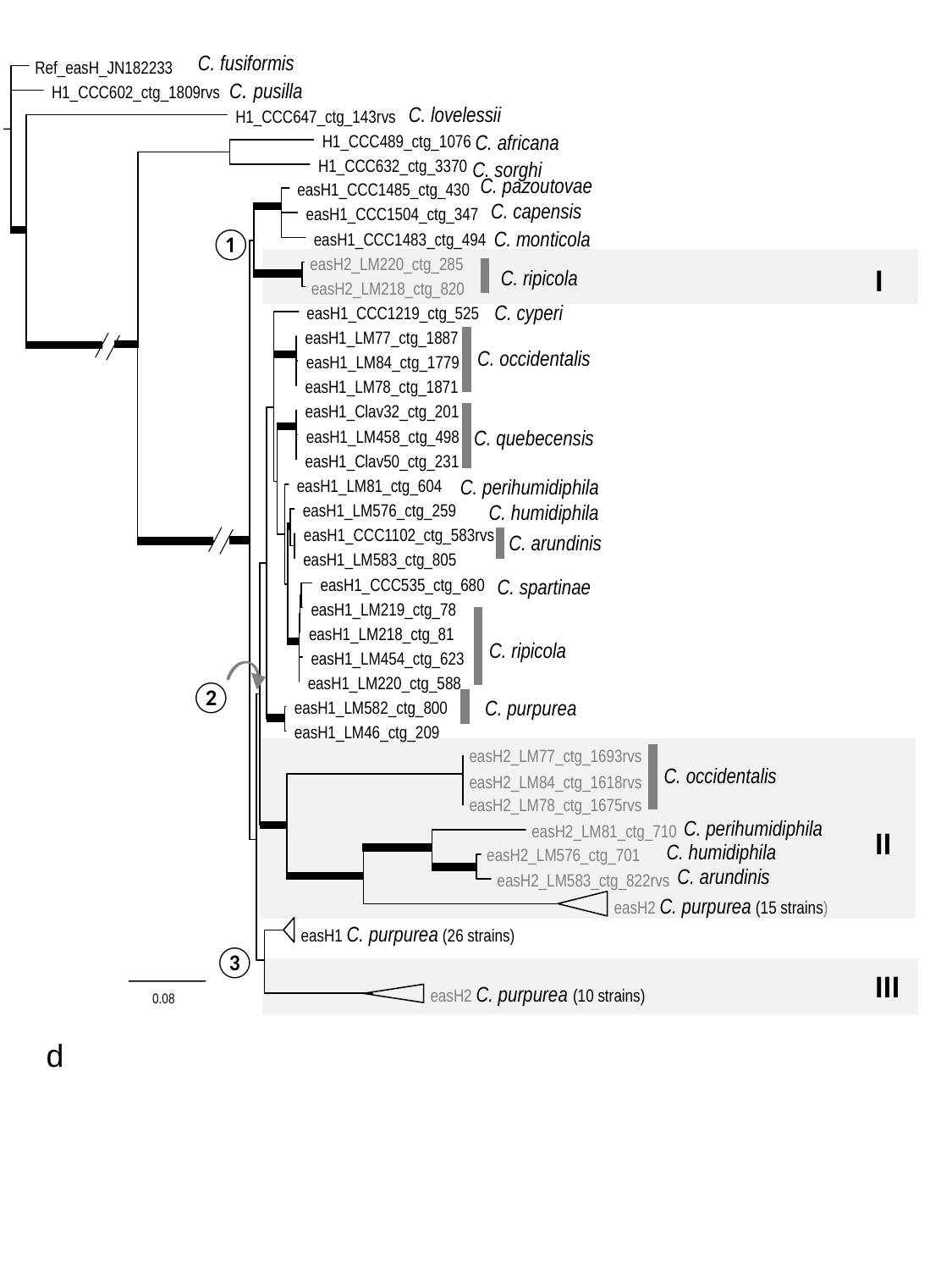

C. fusiformis
Ref_easH_JN182233
C. pusilla
H1_CCC602_ctg_1809rvs
H1_CCC647_ctg_143rvs
H1_CCC489_ctg_1076
H1_CCC632_ctg_3370
C. lovelessii
C. africana
C. sorghi
C. pazoutovae
easH1_CCC1485_ctg_430
C. capensis
easH1_CCC1504_ctg_347
①
C. monticola
easH1_CCC1483_ctg_494
easH2_LM220_ctg_285
I
C. ripicola
easH2_LM218_ctg_820
C. cyperi
easH1_CCC1219_ctg_525
easH1_LM77_ctg_1887
C. occidentalis
easH1_LM84_ctg_1779
easH1_LM78_ctg_1871
easH1_Clav32_ctg_201
C. quebecensis
easH1_LM458_ctg_498
easH1_Clav50_ctg_231
C. perihumidiphila
easH1_LM81_ctg_604
C. humidiphila
easH1_LM576_ctg_259
easH1_CCC1102_ctg_583rvs
C. arundinis
easH1_LM583_ctg_805
easH1_CCC535_ctg_680
C. spartinae
easH1_LM219_ctg_78
easH1_LM218_ctg_81
C. ripicola
easH1_LM454_ctg_623
easH1_LM220_ctg_588
②
C. purpurea
easH1_LM582_ctg_800
easH1_LM46_ctg_209
easH2_LM77_ctg_1693rvs
C. occidentalis
easH2_LM84_ctg_1618rvs
easH2_LM78_ctg_1675rvs
C. perihumidiphila
easH2_LM81_ctg_710
II
C. humidiphila
easH2_LM576_ctg_701
C. arundinis
easH2_LM583_ctg_822rvs
easH2 C. purpurea (15 strains)
easH1 C. purpurea (26 strains)
③
easH2 C. purpurea (10 strains)
III
0.08
d

## Slide 5
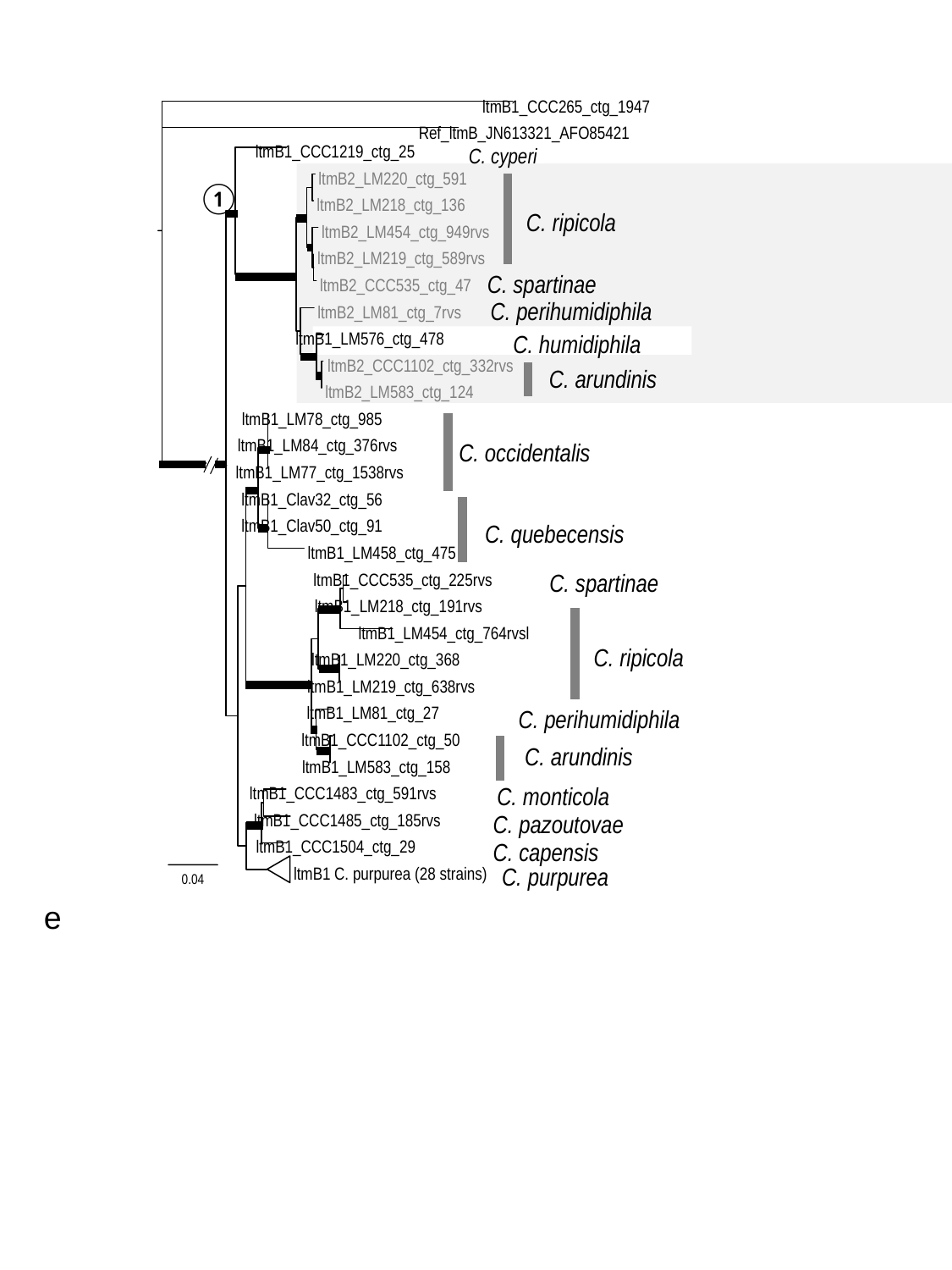

ltmB1_CCC265_ctg_1947
Ref_ltmB_JN613321_AFO85421
ltmB1_CCC1219_ctg_25
ltmB2_LM220_ctg_591
ltmB2_LM218_ctg_136
ltmB2_LM454_ctg_949rvs
ltmB2_LM219_ctg_589rvs
ltmB2_CCC535_ctg_47
ltmB2_LM81_ctg_7rvs
ltmB1_LM576_ctg_478
ltmB2_CCC1102_ctg_332rvs
ltmB2_LM583_ctg_124
ltmB1_LM78_ctg_985
ltmB1_LM84_ctg_376rvs
ltmB1_LM77_ctg_1538rvs
ltmB1_Clav32_ctg_56
ltmB1_Clav50_ctg_91
ltmB1_LM458_ctg_475
ltmB1_CCC535_ctg_225rvs
ltmB1_LM218_ctg_191rvs
ltmB1_LM454_ctg_764rvsl
ltmB1_LM220_ctg_368
ltmB1_LM219_ctg_638rvs
ltmB1_LM81_ctg_27
ltmB1_CCC1102_ctg_50
ltmB1_LM583_ctg_158
ltmB1_CCC1483_ctg_591rvs
ltmB1_CCC1485_ctg_185rvs
ltmB1_CCC1504_ctg_29
ltmB1 C. purpurea (28 strains)
C. cyperi
C. ripicola
①
C. spartinae
C. perihumidiphila
C. humidiphila
C. arundinis
C. occidentalis
C. quebecensis
C. spartinae
C. ripicola
C. perihumidiphila
C. arundinis
C. monticola
C. pazoutovae
C. capensis
C. purpurea
0.04
e

## Slide 6
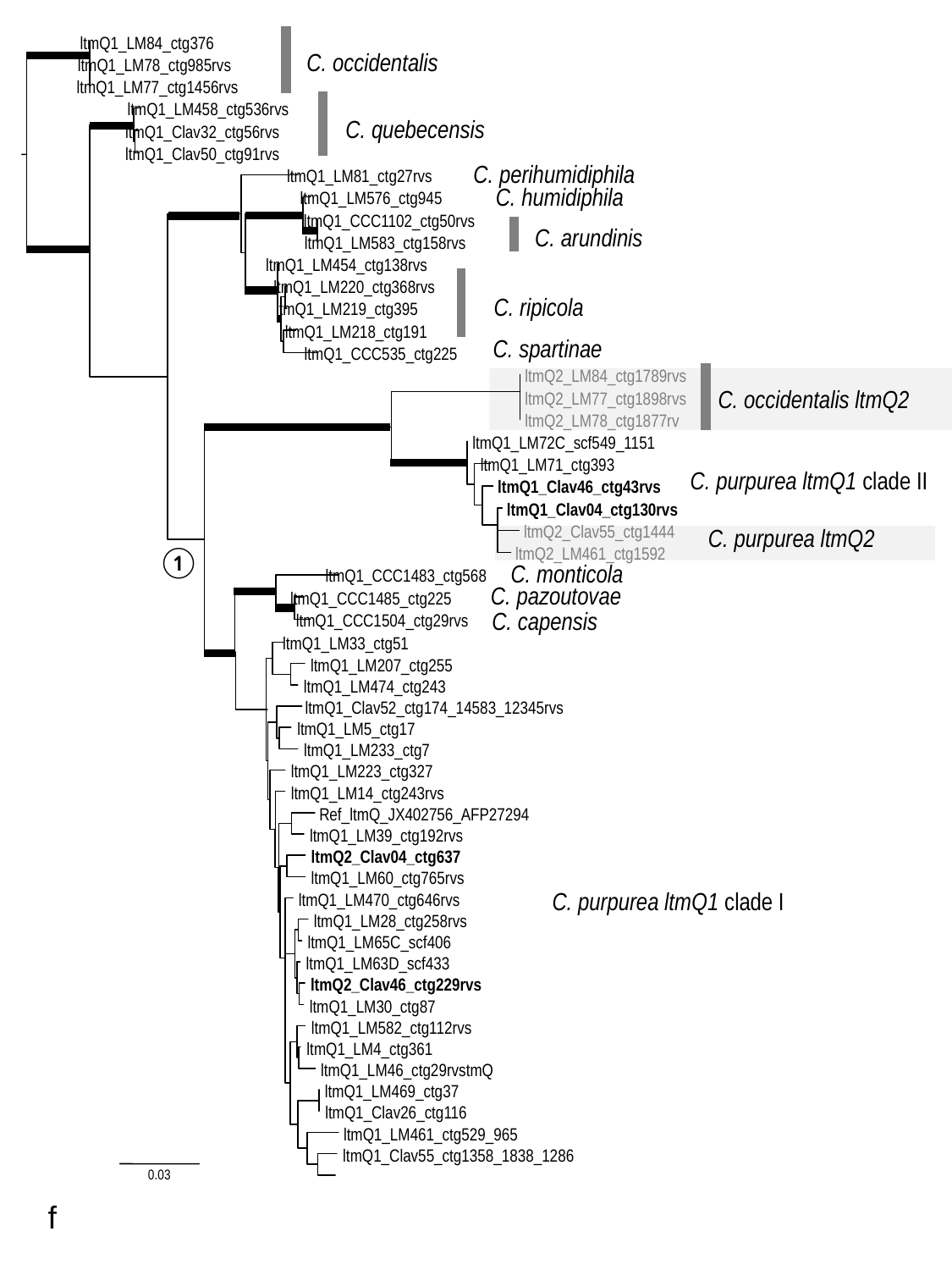

C. occidentalis
ltmQ1_LM84_ctg376
ltmQ1_LM78_ctg985rvs
ltmQ1_LM77_ctg1456rvs
ltmQ1_LM458_ctg536rvs
ltmQ1_Clav32_ctg56rvs
ltmQ1_Clav50_ctg91rvs
ltmQ1_LM81_ctg27rvs
ltmQ1_LM576_ctg945
ltmQ1_CCC1102_ctg50rvs
ltmQ1_LM583_ctg158rvs
ltmQ1_LM454_ctg138rvs
ltmQ1_LM220_ctg368rvs
ltmQ1_LM219_ctg395
ltmQ1_LM218_ctg191
ltmQ1_CCC535_ctg225
ltmQ2_LM84_ctg1789rvs
ltmQ2_LM77_ctg1898rvs
ltmQ2_LM78_ctg1877rv
ltmQ1_LM72C_scf549_1151
ltmQ1_LM71_ctg393
ltmQ1_Clav46_ctg43rvs
ltmQ1_Clav04_ctg130rvs
ltmQ2_Clav55_ctg1444
ltmQ2_LM461_ctg1592
ltmQ1_CCC1483_ctg568
ltmQ1_CCC1485_ctg225
ltmQ1_CCC1504_ctg29rvs
C. quebecensis
C. perihumidiphila
C. humidiphila
C. arundinis
C. ripicola
C. spartinae
C. occidentalis ltmQ2
C. purpurea ltmQ1 clade II
C. purpurea ltmQ2
①
C. monticola
C. pazoutovae
C. capensis
ltmQ1_LM33_ctg51
ltmQ1_LM207_ctg255
ltmQ1_LM474_ctg243
ltmQ1_Clav52_ctg174_14583_12345rvs
ltmQ1_LM5_ctg17
ltmQ1_LM233_ctg7
ltmQ1_LM223_ctg327
ltmQ1_LM14_ctg243rvs
Ref_ltmQ_JX402756_AFP27294
ltmQ1_LM39_ctg192rvs
ltmQ2_Clav04_ctg637
ltmQ1_LM60_ctg765rvs
C. purpurea ltmQ1 clade I
ltmQ1_LM470_ctg646rvs
ltmQ1_LM28_ctg258rvs
ltmQ1_LM65C_scf406
ltmQ1_LM63D_scf433
ltmQ2_Clav46_ctg229rvs
ltmQ1_LM30_ctg87
ltmQ1_LM582_ctg112rvs
ltmQ1_LM4_ctg361
ltmQ1_LM46_ctg29rvstmQ
ltmQ1_LM469_ctg37
ltmQ1_Clav26_ctg116
ltmQ1_LM461_ctg529_965
ltmQ1_Clav55_ctg1358_1838_1286
0.03
f
