# Supplementary material for: Mining Indole Alkaloid Synthesis Gene Clusters from Genomes of 53 Claviceps Strains Revealed Redundant Gene Copies and an Approximate Evolutionary Hourglass Model
Source: Toxins (Basel). 2021 Nov 13;13(11):799. doi: 10.3390/toxins13110799 (PMC8625505; doi:10.3390/toxins13110799)
Supplement: Supplementary file 1 [file toxins-13-00799-s001.zip › !Figure S1_single-copy individual gene trees.pptx]

## Slide 1
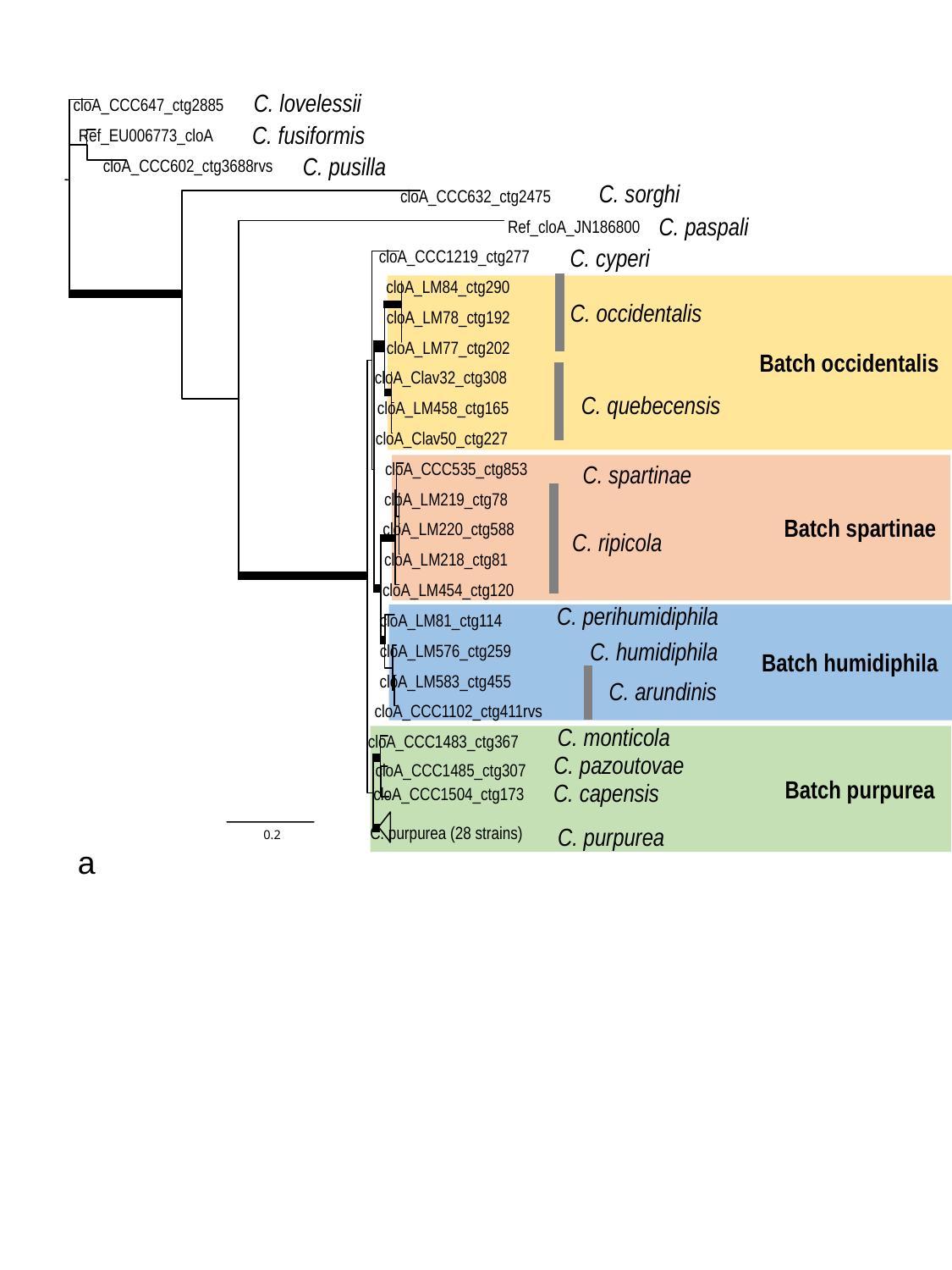

C. lovelessii
cloA_CCC647_ctg2885
C. fusiformis
Ref_EU006773_cloA
C. pusilla
cloA_CCC602_ctg3688rvs
C. sorghi
cloA_CCC632_ctg2475
C. paspali
Ref_cloA_JN186800
C. cyperi
cloA_CCC1219_ctg277
cloA_LM84_ctg290
C. occidentalis
cloA_LM78_ctg192
cloA_LM77_ctg202
cloA_Clav32_ctg308
C. quebecensis
cloA_LM458_ctg165
cloA_Clav50_ctg227
cloA_CCC535_ctg853
C. spartinae
cloA_LM219_ctg78
cloA_LM220_ctg588
C. ripicola
cloA_LM218_ctg81
cloA_LM454_ctg120
C. perihumidiphila
cloA_LM81_ctg114
C. humidiphila
cloA_LM576_ctg259
cloA_LM583_ctg455
C. arundinis
cloA_CCC1102_ctg411rvs
C. monticola
cloA_CCC1483_ctg367
C. pazoutovae
cloA_CCC1485_ctg307
C. capensis
cloA_CCC1504_ctg173
C. purpurea (28 strains)
C. purpurea
0.2
Batch occidentalis
Batch spartinae
Batch humidiphila
Batch purpurea
a

## Slide 2
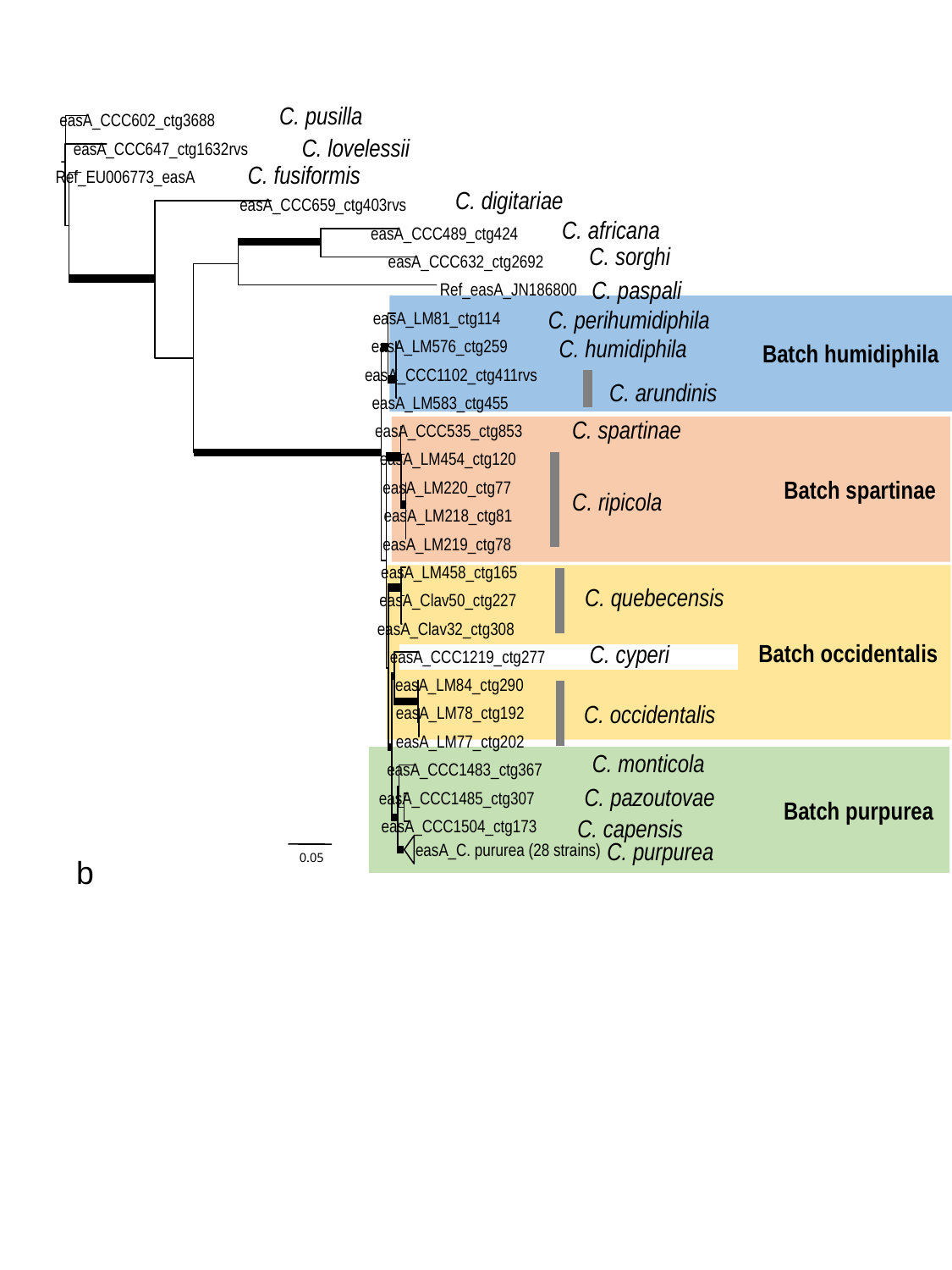

C. pusilla
easA_CCC602_ctg3688
C. lovelessii
easA_CCC647_ctg1632rvs
C. fusiformis
Ref_EU006773_easA
C. digitariae
easA_CCC659_ctg403rvs
C. africana
easA_CCC489_ctg424
C. sorghi
easA_CCC632_ctg2692
C. paspali
Ref_easA_JN186800
Batch humidiphila
C. perihumidiphila
easA_LM81_ctg114
C. humidiphila
easA_LM576_ctg259
easA_CCC1102_ctg411rvs
C. arundinis
easA_LM583_ctg455
C. spartinae
Batch spartinae
easA_CCC535_ctg853
easA_LM454_ctg120
easA_LM220_ctg77
C. ripicola
easA_LM218_ctg81
easA_LM219_ctg78
easA_LM458_ctg165
Batch occidentalis
C. quebecensis
easA_Clav50_ctg227
easA_Clav32_ctg308
C. cyperi
easA_CCC1219_ctg277
easA_LM84_ctg290
C. occidentalis
easA_LM78_ctg192
easA_LM77_ctg202
Batch purpurea
C. monticola
easA_CCC1483_ctg367
C. pazoutovae
easA_CCC1485_ctg307
C. capensis
easA_CCC1504_ctg173
C. purpurea
easA_C. pururea (28 strains)
b
0.05

## Slide 3
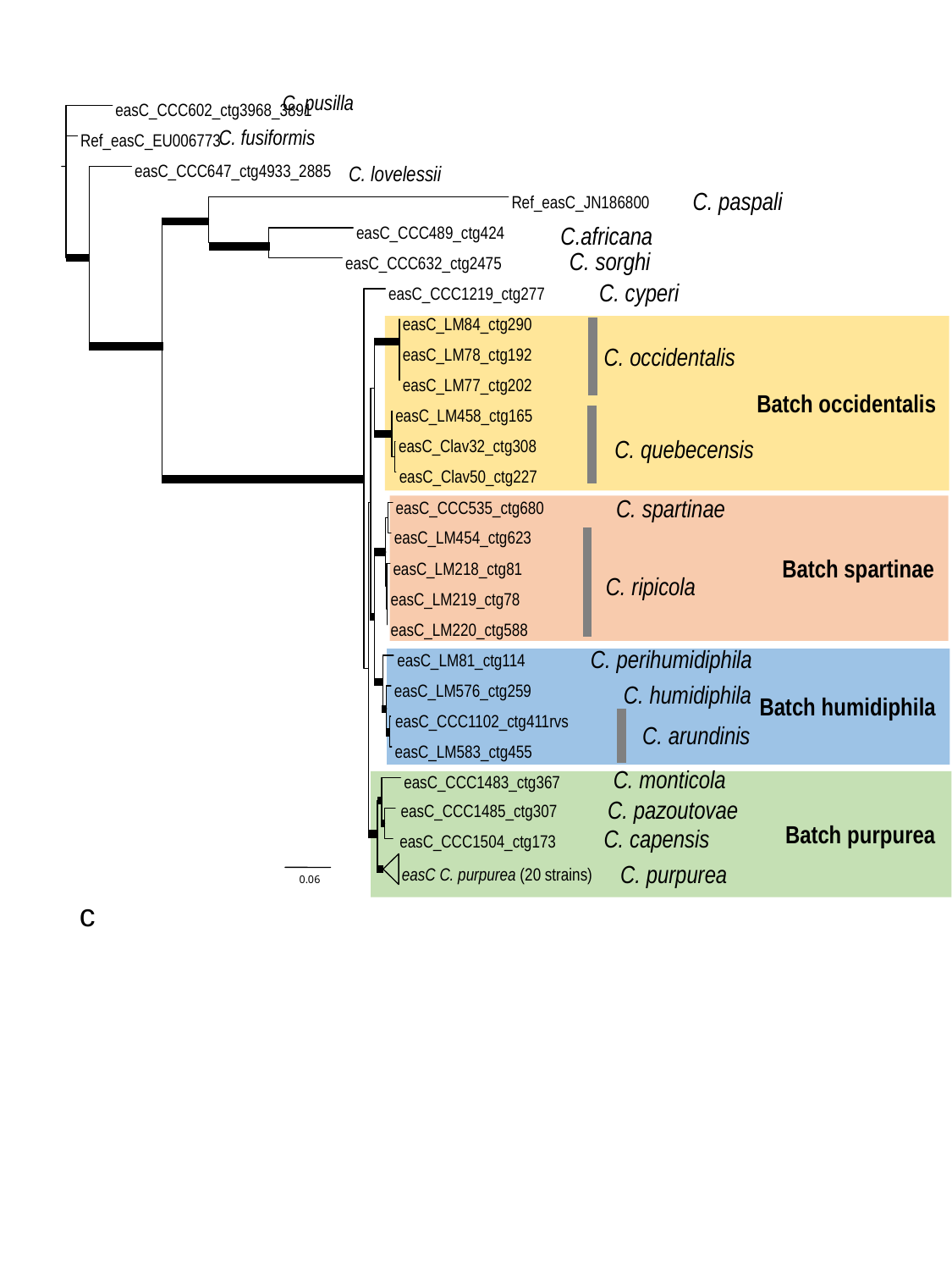

C. pusilla
easC_CCC602_ctg3968_3891
C. fusiformis
Ref_easC_EU006773
easC_CCC647_ctg4933_2885
C. lovelessii
C. paspali
Ref_easC_JN186800
C.africana
easC_CCC489_ctg424
C. sorghi
easC_CCC632_ctg2475
easC_CCC1219_ctg277
easC_LM84_ctg290
easC_LM78_ctg192
easC_LM77_ctg202
easC_LM458_ctg165
easC_Clav32_ctg308
easC_Clav50_ctg227
easC_CCC535_ctg680
easC_LM454_ctg623
easC_LM218_ctg81
easC_LM219_ctg78
easC_LM220_ctg588
easC_LM81_ctg114
easC_LM576_ctg259
easC_CCC1102_ctg411rvs
easC_LM583_ctg455
easC_CCC1483_ctg367
easC_CCC1485_ctg307
easC_CCC1504_ctg173
easC C. purpurea (20 strains)
C. cyperi
C. occidentalis
C. quebecensis
C. spartinae
C. ripicola
C. perihumidiphila
C. humidiphila
C. arundinis
C. monticola
C. pazoutovae
C. capensis
C. purpurea
0.06
c
Batch occidentalis
Batch spartinae
Batch humidiphila
Batch purpurea

## Slide 4
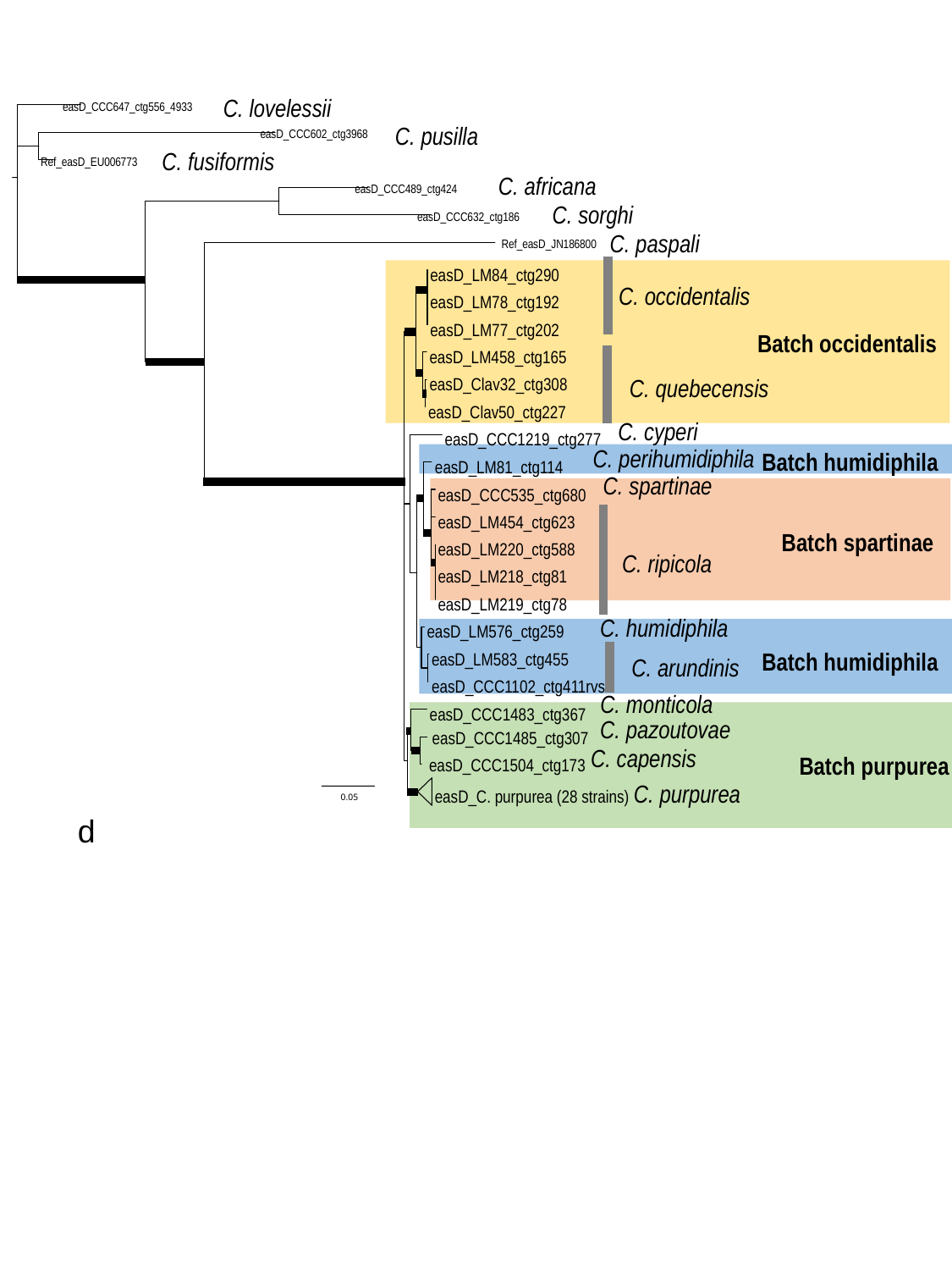

C. lovelessii
easD_CCC647_ctg556_4933
C. pusilla
easD_CCC602_ctg3968
C. fusiformis
Ref_easD_EU006773
C. africana
easD_CCC489_ctg424
C. sorghi
easD_CCC632_ctg186
C. paspali
Ref_easD_JN186800
easD_LM84_ctg290
C. occidentalis
easD_LM78_ctg192
easD_LM77_ctg202
easD_LM458_ctg165
C. quebecensis
easD_Clav32_ctg308
easD_Clav50_ctg227
C. cyperi
easD_CCC1219_ctg277
C. perihumidiphila
easD_LM81_ctg114
C. spartinae
easD_CCC535_ctg680
C. ripicola
easD_LM454_ctg623
easD_LM220_ctg588
easD_LM218_ctg81
easD_LM219_ctg78
C. humidiphila
easD_LM576_ctg259
C. arundinis
easD_LM583_ctg455
easD_CCC1102_ctg411rvs
C. monticola
easD_CCC1483_ctg367
C. pazoutovae
easD_CCC1485_ctg307
C. capensis
easD_CCC1504_ctg173
C. purpurea
easD_C. purpurea (28 strains)
0.05
Batch occidentalis
Batch humidiphila
Batch spartinae
Batch humidiphila
Batch purpurea
d

## Slide 5
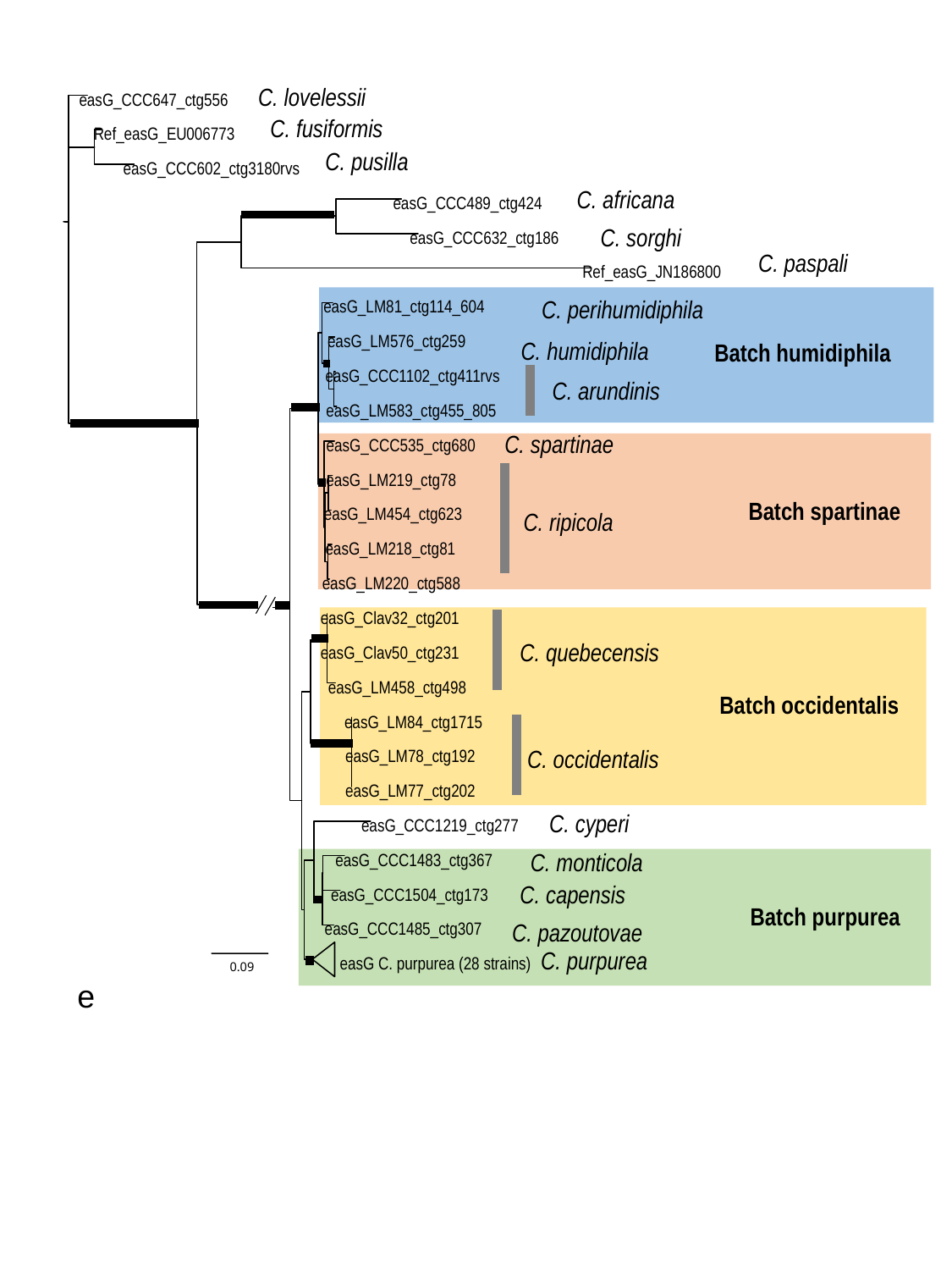

C. lovelessii
easG_CCC647_ctg556
Ref_easG_EU006773
easG_CCC602_ctg3180rvs
easG_CCC489_ctg424
easG_CCC632_ctg186
Ref_easG_JN186800
C. fusiformis
C. pusilla
C. africana
C. sorghi
C. paspali
C. perihumidiphila
easG_LM81_ctg114_604
easG_LM576_ctg259
easG_CCC1102_ctg411rvs
easG_LM583_ctg455_805
easG_CCC535_ctg680
easG_LM219_ctg78
easG_LM454_ctg623
easG_LM218_ctg81
easG_LM220_ctg588
easG_Clav32_ctg201
easG_Clav50_ctg231
easG_LM458_ctg498
easG_LM84_ctg1715
easG_LM78_ctg192
easG_LM77_ctg202
easG_CCC1219_ctg277
easG_CCC1483_ctg367
easG_CCC1504_ctg173
easG_CCC1485_ctg307
easG C. purpurea (28 strains)
C. humidiphila
C. arundinis
C. spartinae
C. ripicola
C. quebecensis
C. occidentalis
C. cyperi
C. monticola
C. capensis
C. pazoutovae
C. purpurea
0.09
Batch humidiphila
Batch spartinae
Batch occidentalis
Batch purpurea
e

## Slide 6
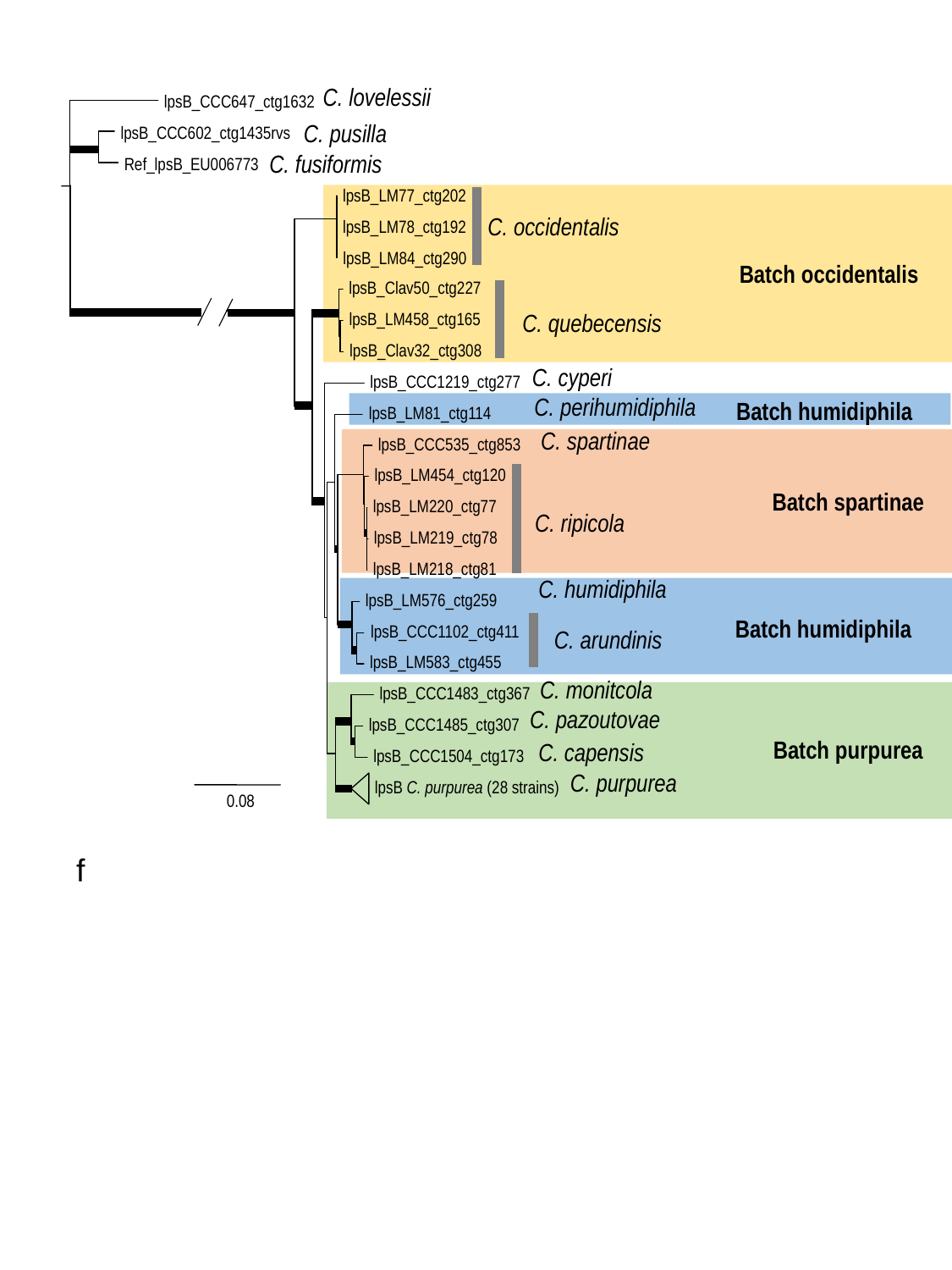

C. lovelessii
lpsB_CCC647_ctg1632
C. pusilla
lpsB_CCC602_ctg1435rvs
C. fusiformis
Ref_lpsB_EU006773
lpsB_LM77_ctg202
lpsB_LM78_ctg192
lpsB_LM84_ctg290
lpsB_Clav50_ctg227
lpsB_LM458_ctg165
lpsB_Clav32_ctg308
lpsB_CCC1219_ctg277
lpsB_LM81_ctg114
lpsB_CCC535_ctg853
lpsB_LM454_ctg120
lpsB_LM220_ctg77
lpsB_LM219_ctg78
lpsB_LM218_ctg81
lpsB_LM576_ctg259
lpsB_CCC1102_ctg411
lpsB_LM583_ctg455
lpsB_CCC1483_ctg367
lpsB_CCC1485_ctg307
lpsB_CCC1504_ctg173
lpsB C. purpurea (28 strains)
Batch occidentalis
C. occidentalis
C. quebecensis
C. cyperi
C. perihumidiphila
Batch humidiphila
C. spartinae
Batch spartinae
C. ripicola
C. humidiphila
Batch humidiphila
C. arundinis
C. monitcola
Batch purpurea
C. pazoutovae
C. capensis
C. purpurea
0.08
f

## Slide 7
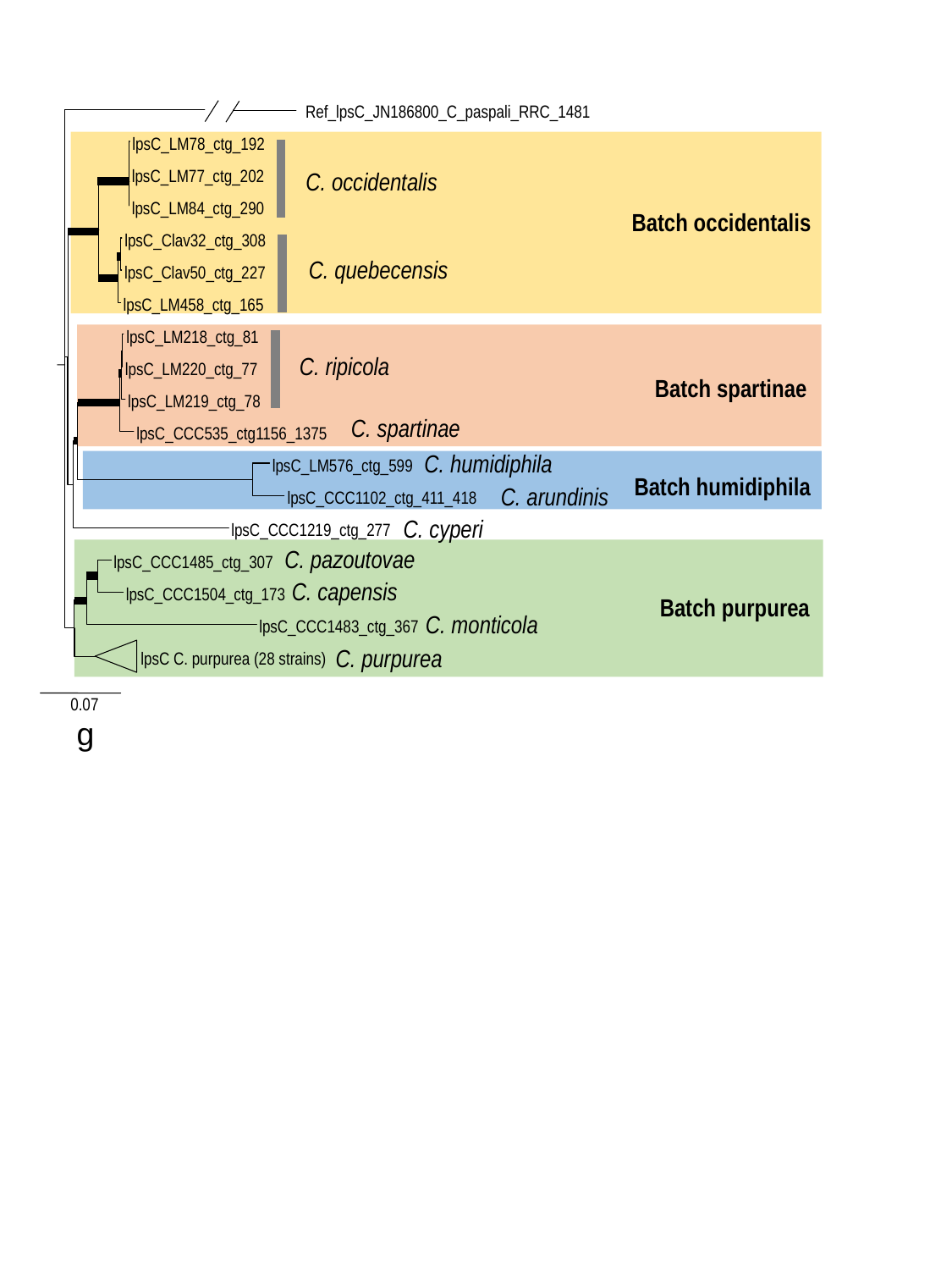

Ref_lpsC_JN186800_C_paspali_RRC_1481
lpsC_LM78_ctg_192
lpsC_LM77_ctg_202
lpsC_LM84_ctg_290
lpsC_Clav32_ctg_308
lpsC_Clav50_ctg_227
lpsC_LM458_ctg_165
lpsC_LM218_ctg_81
lpsC_LM220_ctg_77
lpsC_LM219_ctg_78
lpsC_CCC535_ctg1156_1375
lpsC_LM576_ctg_599
lpsC_CCC1102_ctg_411_418
lpsC_CCC1219_ctg_277
lpsC_CCC1485_ctg_307
lpsC_CCC1504_ctg_173
lpsC_CCC1483_ctg_367
lpsC C. purpurea (28 strains)
0.07
C. occidentalis
C. quebecensis
C. ripicola
C. spartinae
C. humidiphila
C. arundinis
C. cyperi
C. pazoutovae
C. capensis
C. monticola
C. purpurea
Batch occidentalis
Batch spartinae
Batch humidiphila
Batch purpurea
g

## Slide 8
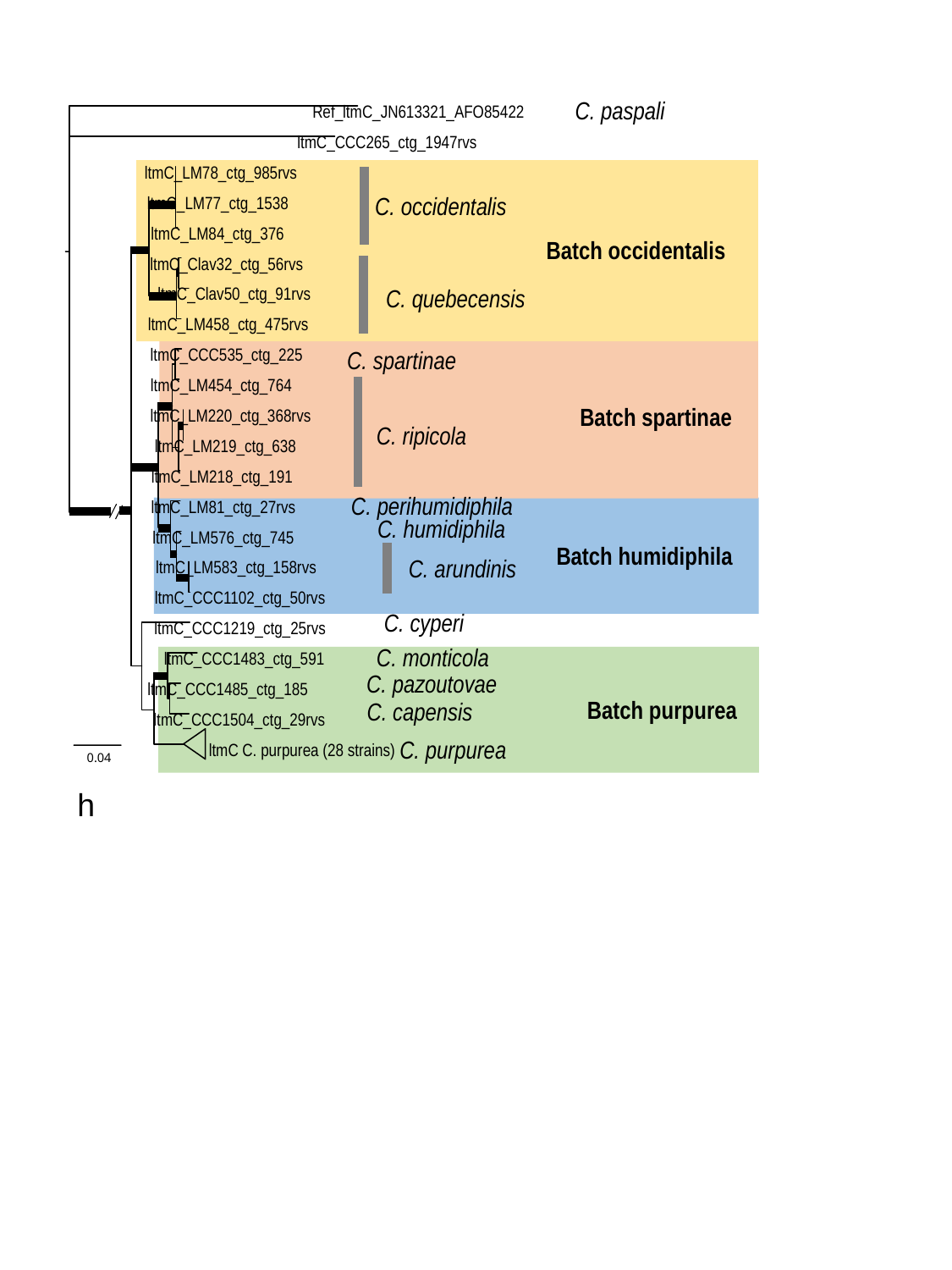

C. paspali
Ref_ltmC_JN613321_AFO85422
ltmC_CCC265_ctg_1947rvs
ltmC_LM78_ctg_985rvs
ltmC_LM77_ctg_1538
ltmC_LM84_ctg_376
ltmC_Clav32_ctg_56rvs
ltmC_Clav50_ctg_91rvs
ltmC_LM458_ctg_475rvs
ltmC_CCC535_ctg_225
ltmC_LM454_ctg_764
ltmC_LM220_ctg_368rvs
ltmC_LM219_ctg_638
ltmC_LM218_ctg_191
ltmC_LM81_ctg_27rvs
ltmC_LM576_ctg_745
ltmC_LM583_ctg_158rvs
ltmC_CCC1102_ctg_50rvs
ltmC_CCC1219_ctg_25rvs
ltmC_CCC1483_ctg_591
ltmC_CCC1485_ctg_185
ltmC_CCC1504_ctg_29rvs
ltmC C. purpurea (28 strains)
0.04
Batch occidentalis
C. occidentalis
C. quebecensis
Batch spartinae
C. spartinae
C. ripicola
C. perihumidiphila
Batch humidiphila
C. humidiphila
C. arundinis
C. cyperi
C. monticola
Batch purpurea
C. pazoutovae
C. capensis
C. purpurea
h

## Slide 9
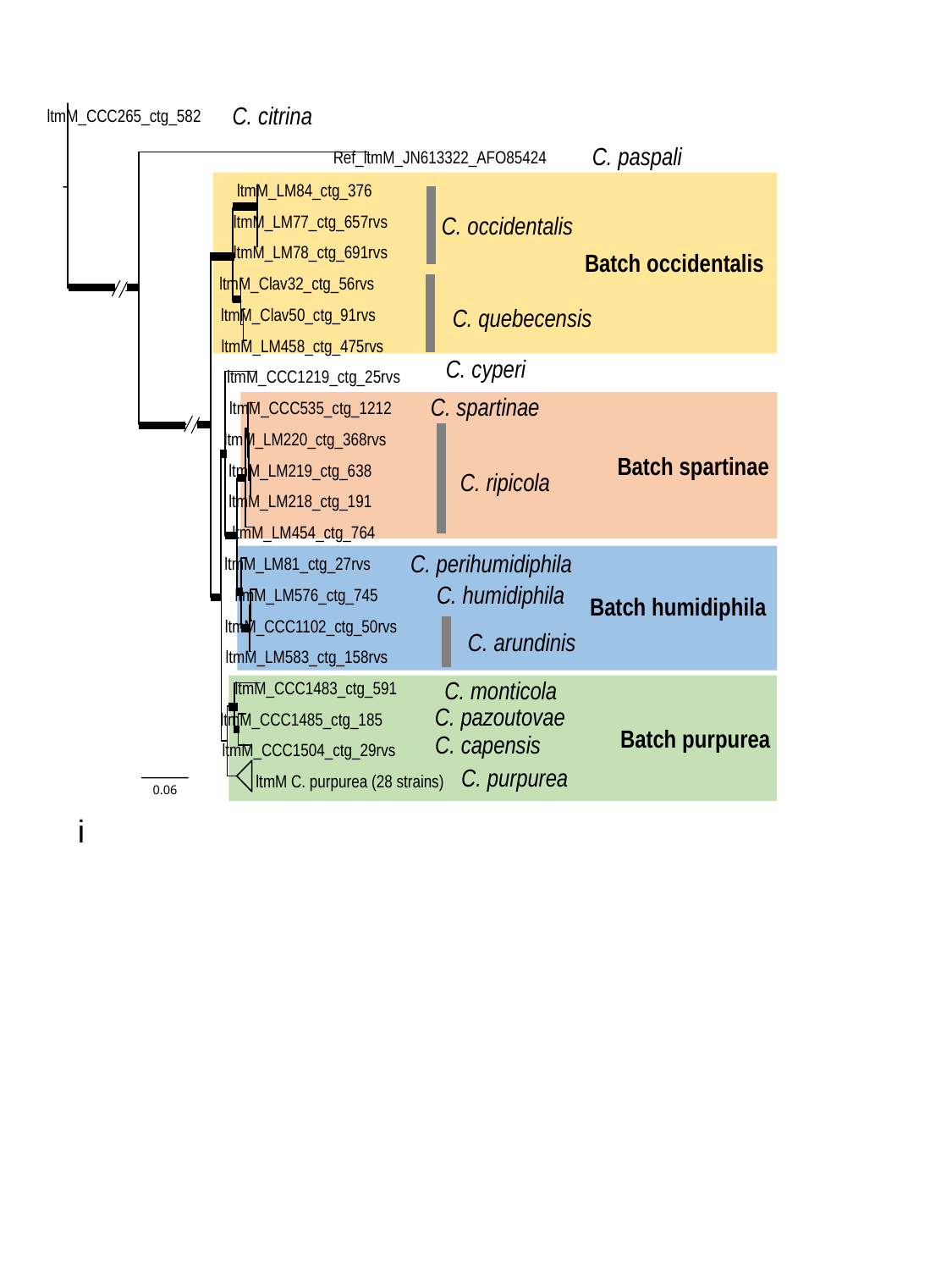

C. citrina
ltmM_CCC265_ctg_582
C. paspali
Ref_ltmM_JN613322_AFO85424
ltmM_LM84_ctg_376
ltmM_LM77_ctg_657rvs
ltmM_LM78_ctg_691rvs
ltmM_Clav32_ctg_56rvs
ltmM_Clav50_ctg_91rvs
ltmM_LM458_ctg_475rvs
ltmM_CCC1219_ctg_25rvs
ltmM_CCC535_ctg_1212
ltmM_LM220_ctg_368rvs
ltmM_LM219_ctg_638
ltmM_LM218_ctg_191
ltmM_LM454_ctg_764
ltmM_LM81_ctg_27rvs
ltmM_LM576_ctg_745
ltmM_CCC1102_ctg_50rvs
ltmM_LM583_ctg_158rvs
ltmM_CCC1483_ctg_591
ltmM_CCC1485_ctg_185
ltmM_CCC1504_ctg_29rvs
ltmM C. purpurea (28 strains)
C. occidentalis
C. quebecensis
C. cyperi
C. spartinae
C. ripicola
C. perihumidiphila
C. humidiphila
C. arundinis
C. monticola
C. pazoutovae
C. capensis
C. purpurea
0.06
Batch occidentalis
Batch spartinae
Batch humidiphila
Batch purpurea
i

## Slide 10
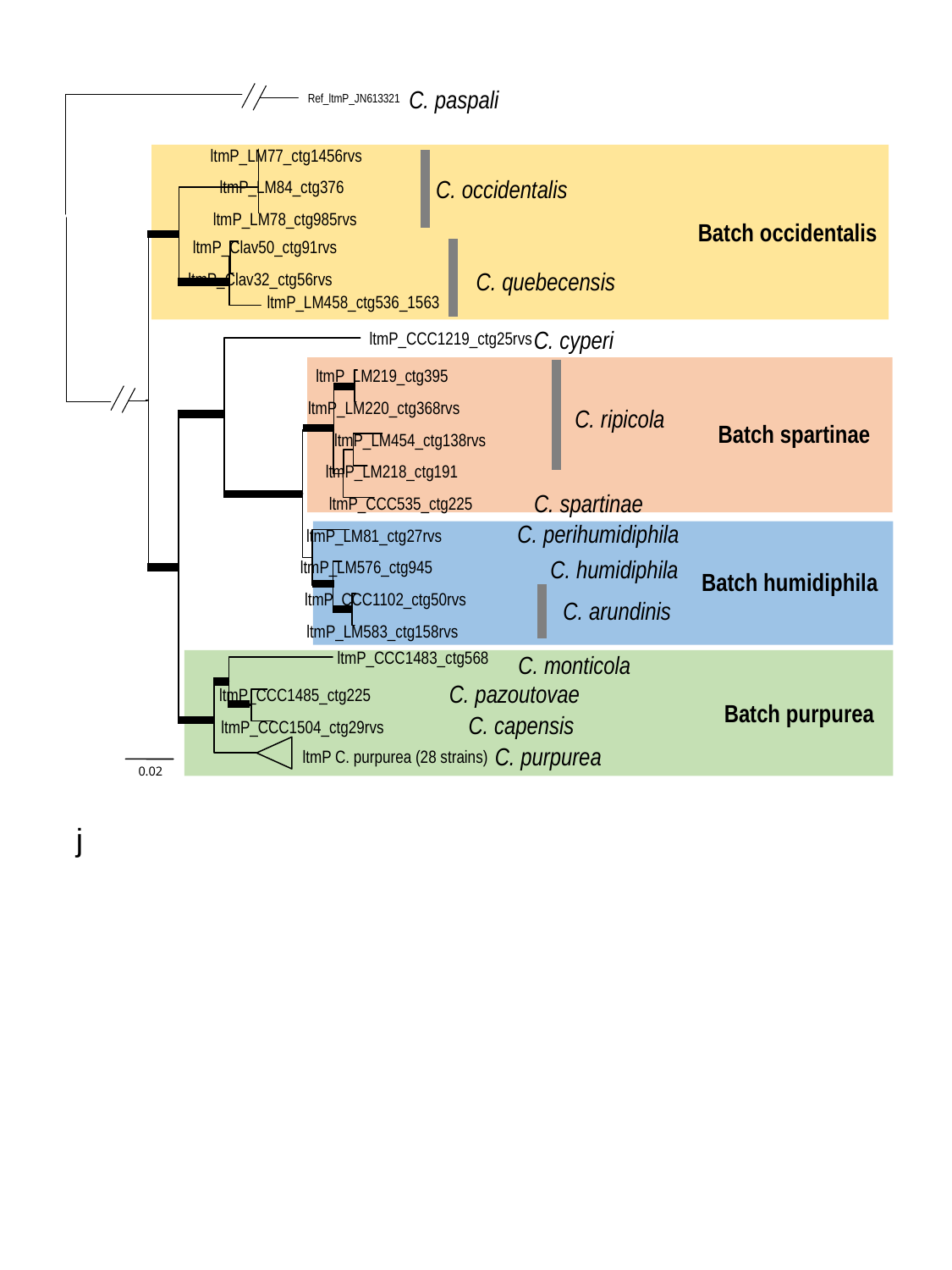

C. paspali
Ref_ltmP_JN613321
ltmP_LM77_ctg1456rvs
ltmP_LM84_ctg376
ltmP_LM78_ctg985rvs
C. occidentalis
ltmP_Clav50_ctg91rvs
ltmP_Clav32_ctg56rvs
ltmP_LM458_ctg536_1563
C. quebecensis
C. cyperi
ltmP_CCC1219_ctg25rvs
C. ripicola
ltmP_LM219_ctg395
ltmP_LM220_ctg368rvs
ltmP_LM454_ctg138rvs
ltmP_LM218_ctg191
C. spartinae
ltmP_CCC535_ctg225
C. perihumidiphila
ltmP_LM81_ctg27rvs
C. humidiphila
ltmP_LM576_ctg945
C. arundinis
ltmP_CCC1102_ctg50rvs
ltmP_LM583_ctg158rvs
ltmP_CCC1483_ctg568
C. monticola
C. pazoutovae
ltmP_CCC1485_ctg225
C. capensis
ltmP_CCC1504_ctg29rvs
C. purpurea
ltmP C. purpurea (28 strains)
0.02
Batch occidentalis
Batch spartinae
Batch humidiphila
Batch purpurea
j

## Slide 11
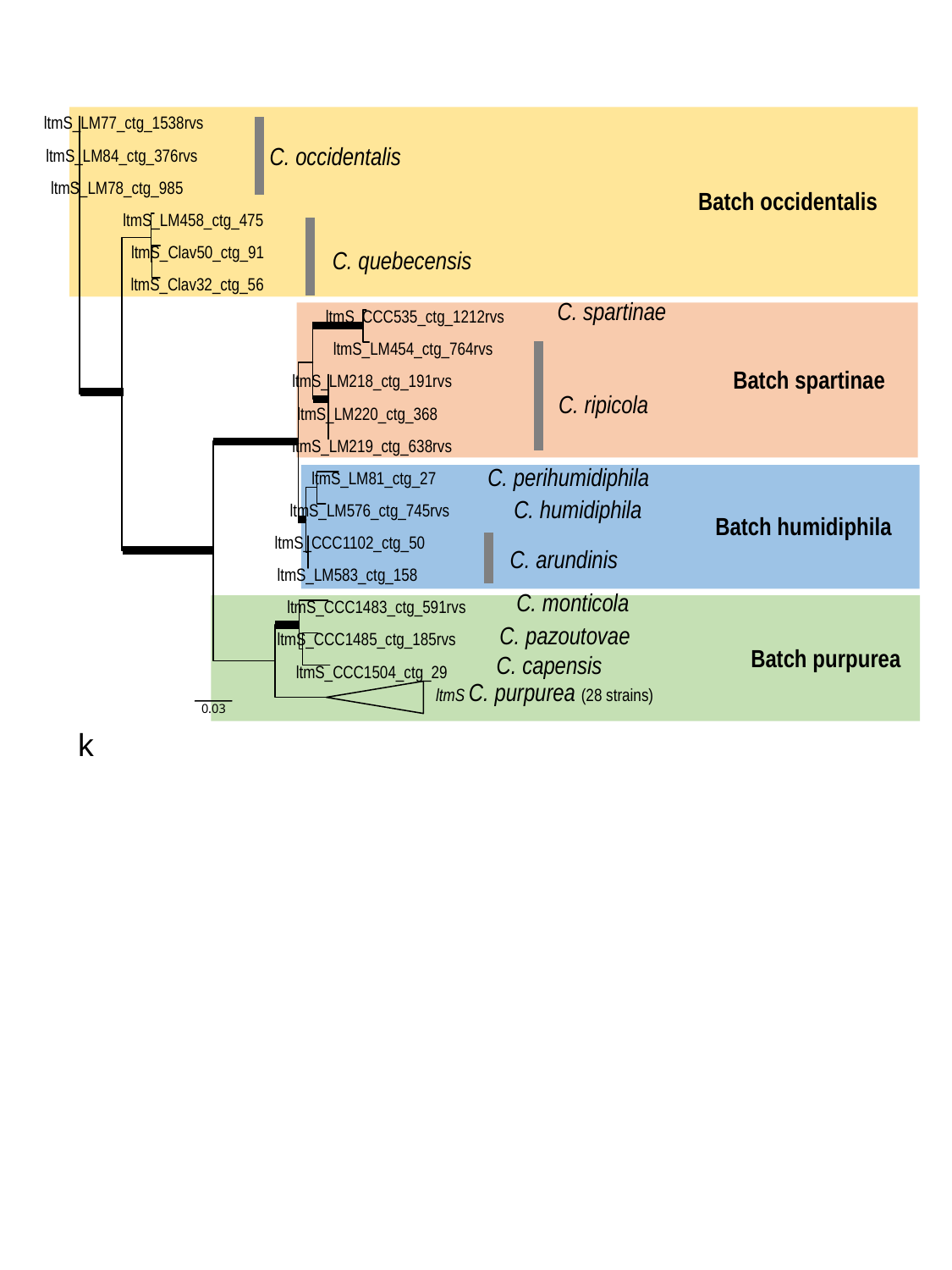

Batch occidentalis
ltmS_LM77_ctg_1538rvs
C. occidentalis
ltmS_LM84_ctg_376rvs
ltmS_LM78_ctg_985
ltmS_LM458_ctg_475
C. quebecensis
ltmS_Clav50_ctg_91
ltmS_Clav32_ctg_56
C. spartinae
ltmS_CCC535_ctg_1212rvs
ltmS_LM454_ctg_764rvs
ltmS_LM218_ctg_191rvs
C. ripicola
ltmS_LM220_ctg_368
ltmS_LM219_ctg_638rvs
C. perihumidiphila
ltmS_LM81_ctg_27
C. humidiphila
ltmS_LM576_ctg_745rvs
ltmS_CCC1102_ctg_50
C. arundinis
ltmS_LM583_ctg_158
C. monticola
ltmS_CCC1483_ctg_591rvs
C. pazoutovae
ltmS_CCC1485_ctg_185rvs
C. capensis
ltmS_CCC1504_ctg_29
ltmS C. purpurea (28 strains)
0.03
Batch spartinae
Batch humidiphila
Batch purpurea
k
